# Set similarity modulates object tracking in dynamic environments

**DOI:** 10.3758/s13414-018-1559-y

**Published:** 2018-07-02

**Authors:** Sibel Akyuz, Jaap Munneke, Jennifer E. Corbett

**Affiliations:** 1 Department of Psychology, Osmaniye Korkut Ata Universitesi, Osmaniye, Turkey; 2 Aysel Sabuncu Brain Research Center, Bilkent University, Ankara, Turkey; 3 Interdisciplinary Neuroscience Program, Bilkent University, Ankara, Turkey; 4 College of Health and Life Sciences, Division of Psychology, MJ-122, Brunel University London, Kingston Lane, Uxbridge, London UB8 3PH UK; 5 Center for Cognitive Neuroscience, Brunel University London, London, UK

**Keywords:** Grouping and segmentation, Perceptual organization, Attention

## Abstract

Based on the observation that sports teams rely on colored jerseys to define group membership, we examined how grouping by similarity affected observers’ abilities to track a “ball” target passed between 20 colored circle “players” divided into two color “teams” of 10 players each, or five color teams of four players each. Observers were more accurate and exerted less effort (indexed by pupil diameter) when their task was to count the number of times any player gained possession of the ball versus when they had to count only the possessions by a given color team, especially when counting the possessions of one team when players were grouped into fewer teams of more individual members each. Overall, results confirm previous reports of costs for segregating a larger set into smaller subsets and suggest that grouping by similarity facilitates processing at the set level.

Even though the image falling on the retina is constantly changing as we interact within our dynamic surroundings, we nonetheless maintain a seemingly stable, high-resolution impression of the chaotic world around us. The visual system responsible for creating this representation is limited to processing only a few salient objects in detail at any given moment (e.g., Luck & Vogel, 1997). However, there is much evidence to suggest that the remaining majority of information in each glance plays a major role in forming and sustaining our notion of spatiotemporal completeness. Along these lines, the visual system relies on a default set of heuristic regularities in the external environment to guide the formation of initial perceptual chunks, which pragmatically constrain further processing (e.g., Wertheimer, 1938). These perceptual chunks likely consist of sparse statistical representations of redundant information (Corbett, 2017). Toward furthering our understanding of how the limited-capacity visual system relies on such higher order regularities to organize and segregate information in dynamic environments, we examined how observers utilized grouping by similarity to track a target within a display composed of one or more subsets of objects. We modeled our paradigm after the real-world scenario of viewing a soccer match in which observers have to actively track a ball passed within and between color-defined groups (i.e., teams wearing different colored jerseys). More specifically, we investigated whether higher order grouping modulates observers’ abilities to track who has possession of the ball as it is dynamically passed between players.

On the one hand, traditional limited-capacity models of perception and attention that propose that the visual system can only keep track of a very limited number of objects at once (e.g., Pylyshyn & Storm, 1988) predict that grouping players into different teams should have no effect on observers’ abilities to track the soccer ball. On the other hand, in line with proposals that there is no fixed limit to attentional processing (e.g., Davis, Welch, Holmes, & Shepherd, 2001), such that attention is flexibly allocated according to factors such as task demands and stimulus discriminability (e.g., Alvarez & Franconeri, 2007), grouping players into different color teams may differentially affect the manner in which observers are able to track the ball. Along these lines, there is mounting evidence that the manner in which information is organized affects the extent to which observers can attend, memorize, and act on this information. For example, when the spatial location of an object that was part of a set of objects grouped by proximity or connectedness in a study display was precued, participants were more accurate at recalling whether other members of the same Gestalt group were present in a subsequent test display (Woodman, Vecera, & Luck, 2003). Similarly, Brady and Tenenbaum (2013) reported that grouping by similarity and proximity improved change-detection performance compared with performance with arrays that did not contain Gestalt grouping cues. More recently, Corbett (2017) reported that when observers viewed study displays of heterogeneously sized circles organized into two different Gestalt-defined mean size groups, followed by test displays of a subset of homogeneously sized circles and were asked to adjust the test circles to the remembered sizes of the corresponding circles in the study displays, they made more similar (correlated) errors for test circles in the same Gestalt-defined groups. Their adjustments of physically identical test circles were also biased toward the mean sizes of the corresponding Gestalt-defined group mean sizes.

In addition to these behavioral effects, several studies have found evidence that Gestalt grouping reduces the amount of neural resources needed to maintain sets of items in visual short-term memory (VSTM). For example, Xu and Chun (2007) demonstrated lower inferior parietal sulcus (IPS) activation (an inverse index of the number of objects that are currently represented in VSTM) for sets of shapes grouped by common region compared with ungrouped displays of shapes. Gao et al. (2011) demonstrated a similar benefit of grouping by similarity for sets of same color objects such that another index of VSTM capacity, the amplitude of the contralateral delay activity (CDA) event-related potential (ERP) component was similar to the CDA amplitude evoked by one object of the same color and lower than the amplitude of the CDA evoked by four distinctly colored objects. Taken together, these findings provide strong support for the proposal that observers rely on higher order structure to perceptually group and segregate visual information. Furthermore, even when observers are not explicitly aware of high-level grouping heuristics, the overall configuration of background information can nonetheless exert a strong influence on how objects within the current focus of attention are processed and perceived (e.g., Moore & Egeth, 1997). As such, grouping and segregating do not solely rely on processing low-level visual information at the individual object level; they also implicitly depend on higher order visual information.

Whereas there is mounting evidence to suggest that grouping objects reduces the amount of resources needed for encoding and increases the amount of information that can be stored in VSTM (e.g., Brady & Tenenbaum, 2013; Corbett, 2017; Gao et al., 2011; Peterson, Gozenman, Arciniega, & Berryhill, 2015; Woodman et al., 2003; Xu & Chun, 2007), it remains unclear whether such benefits arise from an increased ability to process more individual objects or from a facilitation of processing over entire groups of objects. Gestalt grouping can modulate what is considered to be the basic unit of processing, from single objects to collections or groups of objects (e.g., Scholl, 2001; Yantis, 1992), such that information about an entire group can be accessed based on a single feature value (e.g., all red items; Huang, 2010; Huang & Pashler, 2007). Along these lines, several studies from the perceptual averaging literature have demonstrated evidence that information about an entire set of objects can be represented even when individual items cannot, but there is likely a cost for representing multiple sets in parallel (Attarha & Moore, 2015; Attarha, Moore, & Vecera, 2014; Brand, Oriet, & Tottenham, 2012; Oriet & Brand, 2013). Taken together, these results suggest a fixed capacity for processing average properties of multiple sets of objects, but an unlimited capacity for averaging over these subgroups.

To determine whether grouping (by similarity of color) helps observers track interactions between individual objects or facilitates processing over an entire group, we conducted an experiment designed to mimic circumstances similar to a real-world soccer match. Observers viewed dynamic displays of different “teams” wearing different colored “jerseys” passing a ball, and their task was either to track the passes between all “players,” or only players on a prespecified color team. In addition to measuring behavioral accuracy, we measured pupil diameter to gain further insight into the effects of grouping on the cognitive demands (i.e., mental resources) of processing interactions between multiple objects (e.g., Alnæs et al., 2014). Prior work has shown that pupil diameter is an accurate indicator of the mental effort invested in visual search and counting tasks, such that pupil size increases with search difficulty and set size (Porter, Troscianko, & Gilchrist, 2007). Along these lines, measuring pupil diameter in the current tracking task provides additional insight into how the mental effort required to track the ball target may be modulated as a function of manner in which individual objects are organized. To the extent that color-similarity grouping facilitates observers in tracking the interactions between subsets of individual objects, they should have smaller pupil diameters and be more accurate when counting the possessions of one color group among many different groups composed of only a few objects each, compared with when counting the possessions of one color group among fewer groups with more individual objects in each group.

Given that larger pupil diameters tend to reflect more effortful top-down task demands, and top-down attention has been shown to facilitate performance in similar tasks such as visual search (e.g., Chun & Jiang, 1999), we were also interested in whether having a specific top-down set alters the influence of grouping. For example, the manner in which observers rely on the grouping structure of displays may be modulated by the task they are attempting to perform. More specifically, observers may rely on the grouping structure of displays differently when their task is to count all of the possessions of the ball regardless of team membership than when they are instructed to count only the possessions by a prespecified color team. In line with previous perceptual averaging findings (e.g., Attarha & Moore, 2015; Attarha et al., 2014), if observers have a fixed capacity for processing multiple subsets but unlimited capacity for processing over all sets, then performance should be better when their task is to count the interactions between all the players in the entire display versus when they are only tasked to attend the interactions between a specified subset.

## Method

### Participants

Twenty-nine students at Bilkent University (20 female, mean age = 19.8 years) voluntarily participated in an experimental session lasting approximately 120 minutes, for either course credit or monetary compensation. All participants had normal or corrected-to-normal vision. All experimental procedures and protocols were approved by Bilkent University’s Ethics Committee.

### Task

**Fig. 1:**
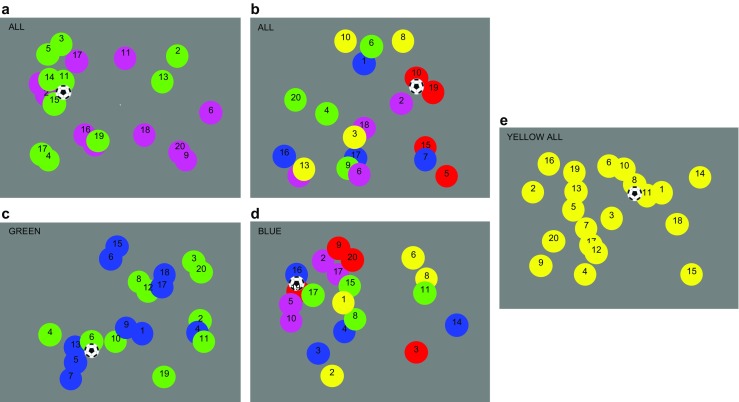
On each trial, participants counted the number of times any of the players (CountAll) or a player from a prespecified color team (CountTeam) gained possession of the ball. There were five possible conditions: **a** CountAll2Teams: Players were grouped into two teams of 10 players each, and participants had to count all of the possessions during the trial. **b** CountAll5Teams: Players were grouped into five teams of four players each, and participants had to count all of the possessions. **c** CountTeam2Teams: Players were grouped into two teams of 10 players, and participants had to count only the possessions of a prespecified color team (e.g., GREEN). **d** CountTeam5Teams: Players were grouped into five teams of four players, and participants had to count only the possessions of a prespecified color team (e.g., BLUE). **e** 1Team: Players were all the same color, and participants had to count all of the possessions (e.g., YELLOW ALL). (Color figure online)

The task was designed to mimic a soccer match, with different colored “teams” of individual circle “players” passing a soccer ball from one player to another (see Fig. 1). On each trial, participants’ task was either to count how many times any of the players gained possession of the ball (CountAll) or how many times a team with a prespecified color gained possession of the ball (CountTeam). There could be one team of 20 players (1Team—baseline condition), two teams of 10 players (2Teams), or five teams of four players each (5Teams). A possession occurred whenever any player (in the CountAll condition) or a member of the given color team (in the CountTeam condition) received the ball from any other player (on the same or a different color team), but not when the same player moved with the ball.

### Apparatus

Stimuli were presented on a Dell PC on a gray (midway between black and white) background on a 21-in. NEC monitor with a refresh rate of 60 Hz and a resolution of 1600 × 1200 pixels. MATLAB software (Version 2016a; The MathWorks, Natick, MA) in conjunction with the Psychophysics Toolbox 3 (Brainard, 1997; Pelli, 1997) controlled stimulus presentation and data collection. Participants were seated 57 cm from the center of the screen, with their heads restrained by a chin rest, such that 1° of visual angle corresponded to 37 pixels. Participants’ pupil diameter was recorded by an Eye-Trac 6 system (Applied Science Laboratories, Bedford, MA). The tracker monitored the right eye at a sampling rate of 60 Hz.^1^

### Stimuli and procedure

On each trial, participants viewed displays of 20 colored circle players passing a soccer ball in one of five possible conditions. In the CountAll2Teams condition, the 20 players were divided into two color teams of 10 players each, and participants were instructed to count all of the possessions regardless of the color teams to which the individual players belonged (“ALL”; see Fig. 1a). Similarly, in the CountAll5Teams condition, the 20 players were divided into five color teams of four players each, and participants were instructed to count all the possessions regardless of color team membership (“ALL”; see Fig. 1b). In the CountTeams2Teams condition, the players were again divided into two teams of 10 players each, but participants were instructed to count only the possessions within a color-defined team (e.g., “GREEN”; see Fig. 1c), and in the CountTeam5Teams condition, the players were divided into five teams of four players each, and participants were instructed to count only the possessions within a color-defined team (e.g., “BLUE”; see Fig. 1d). Finally, in the baseline 1Team condition, all players were presented in the same color, and participants were instructed to count all of the possessions (e.g., “YELLOW ALL”; see Fig. 1e). The order of conditions was pseudorandomized within each block.

Participants initiated each trial with a space bar press. Next, the specific task instructions (e.g., “GREEN”) appeared in the center of the screen. On each trial, the specific color (in the 1Team condition), or the colors of the teams (in the CountAll and CountTeam conditions) were randomly selected. In the CountTeam conditions, the “tracked team” was randomly selected from the subset of the trial’s color teams. After the participant pressed the space bar again, the 20 players appeared, passing the soccer ball for a randomly selected duration between 12 and 14 s (in 500-ms steps), with the specific instructions (e.g., “GREEN”) continuously present in the top left corner of the display. Subsequently, the text “How many possessions?” appeared in 32-point black font in the center of the screen, prompting participants to enter a two-digit response (e.g., “08”) using the top row of numbers on the computer keyboard.

Each trial began with the 20 players randomly positioned within 25 possible starting locations, arranged in an imaginary square 5 × 5 grid that was centered in the middle of the monitor and subtended approximately 17.5° of visual angle. Individual players were 2° (diameter) circles presented in one of five possible colors: Red = [255 0 0]; Green = [0 255 0]; Blue = [0 0 255]; Yellow = [255 255 0]; Magenta = [255 0 255]. A 24-point black number was centered in the top of each circular player. At the start of each trial, the numbers 1 through 20 were randomly assigned over the players in each separate color team, and the number associated with a given player remained constant for the duration of the trial. The soccer ball was a black and white JPEG image 3° in diameter, and was always presented at the player’s “feet” (centered with the player horizontally, and positioned vertically starting from the center of the player and extending down such that the ball never overlapped with the possessing player’s number).

**Fig. 2:**
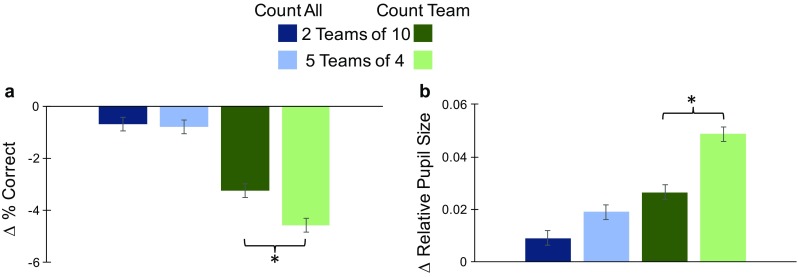
Results (*n* = 29). **a** % correct: When counting the possessions within a specific team (CountTeam), participants were significantly more accurate when there were two teams of 10 players each (CountTeam2Teams) versus when there were five teams of four players each (CountTeam5Teams). **b** Pupil diameter: Participants’ relative pupil diameters were significantly smaller when counting the possessions of a specific color team (CountTeam) when there were two teams of 10 players (CountTeam2Teams) compared with when there were five teams of four players each (CountTeam5Teams). Error bars represent 95% within-subjects confidence intervals using the mean squared error of the two-way interactions in the corresponding ANOVAs (Loftus & Masson, 1994). Asterisks represent significant planned comparisons with *p* < .05

Each trial started with a random player possessing the ball. Participants were explicitly instructed that this starting position should not be counted in their total as “gaining possession” of the ball. Throughout the trial, at random intervals from 400 ms to 600 ms (in 50-ms steps), each player had a 50% chance of changing location. A player that changed location immediately reappeared at a new location offset by randomly selected amounts in the *x* and *y* directions, from −2° to +2° in 0.05° steps. Note that although the soccer ball was never occluded, players could overlap with one another (see Fig. 2) similar to circumstances in a real-world soccer match. The ball changed players on approximately 90% of the intervals within a given trial. To better equate performance across the CountTeam2Teams and CountTeam5Teams conditions, one of the color teams was randomly selected at the start of the trial (this was the “tracked team” in the CountTeam conditions), and players on this team received approximately 50% of the possessions during the trial. On all trials, the movements of the players and the ball were restricted within an imaginary boundary approximately 3.35° (the size of a single player + 50 pixels) from the horizontal and vertical edges of the screen.

The entire experimental session consisted of eight blocks of 30 trials each, for a total of 240 trials. There were six trials of each of the five conditions presented in pseudorandom order in each block, for a total of 48 trials per condition. Participants were given short breaks before calibrating the eye tracker between each block, and a longer (3 to 5 min) break between the fourth and fifth blocks.

Participants were presented with written, illustrated instructions in their language of choice (Turkish or English) at the start of each session. The instructions stressed accuracy in counting the number of task-relevant possessions. We established a performance criterion in order to assure that participants were performing the task as instructed. Participants failed a given block if their average absolute error in counting possessions was greater than five (i.e., on average five too many or too few possessions were reported). Before beginning the experimental blocks, participants completed a block of 10 practice trials. If they failed the practice block, they were allowed to repeat it once. If they failed a second time, they were dismissed from the experiment. Participants were allowed to repeat only one experimental block during the entire course of the session. Upon any subsequent failure in an experimental block, the participant was dismissed from the remainder of the experiment. A total of two participants (not included in the above description of participant demographics or in any of the analyses) were dismissed for failing to meet this criterion.

### Analysis

Eight additional participants (not included in the above description of participant demographics) were excluded from further analysis because the eye tracker lost the pupil for four or more samples on more than 10% of trials in a single block. For the remaining participants’ data, trials with absolute errors greater than 10, responses of zero, and trials for which pupil size could not be consistently measured or interpolated were excluded from further analysis (less than 2% of trials for any participant). We used the 1Team condition where there was only one color team, and participants had to count all of the possessions as a baseline against which to compare performance when participants were counting the possessions of all players in two or five different color teams (CountAll conditions) or were counting only the possessions by one of the two or five different teams (CountTeam conditions). Accordingly, for each participant, we subtracted the mean of performance in the 1Team condition from the mean of each of the other four conditions (CountAll2Teams, CountAll5Teams, CountTeam2Teams, and CountTeam5Teams). The resultant means of the four conditions are illustrated relative to this zero/baseline measure of performance on the *y*-axis in Fig. 2, and the raw conditional means and standard deviations for percentage correct and pupil diameter in all five conditions (CountAll2Teams, CountAll5Teams, CountTeam2Teams, CountTeam5Teams, and 1Team) are listed in Table 1. As our primary hypothesis concerned potential differences in performance when there were two groups of 10 players compared with when there were five groups of four players, we conducted a 2(CountTask: CountAll/CountTeam) × 2(NTeams: 2Teams/5Teams) ANOVA on the relative data followed by two planned comparisons between the two team sizes (2Teams/5Teams) in each CountTask for each of the two dependent measures (% correct and pupil diameter).

**Table 1:** Raw (baseline not subtracted) percentage correct (top) and pupil diameter (bottom) means and standard deviations (in parenthesis) in the five CountAll2Teams, CountAll5Teams, CountTeam2Teams, CountTeam5Teams, and 1Team conditions

	CountAll2Teams	CountAll5Teams	CountTeam2Teams	CountTeam5Teams	1Team
% correct	93.89 (−5.48)	93.79 (−5.41)	91.32 (−9.74)	90.02 (−11.07)	94.58 (−5.34)
Pupil diameter	0.997 (−0.099)	1.003 (−0.092)	1.011 (−0.093)	1.033 (−0.089)	0.984 (−0.108)

## Results

### Percentage correct

We calculated percentage correct on each trial by dividing the value of the actual number of possessions (NPos) minus the absolute error on each trial (AbsError) by the actual number of possessions (NPos), and then multiplying by 100, using the following formula:

$$ \%\mathrm{Correct}=\left(\left(\mathrm{NPos}-\mathrm{AbsError}\right)/\mathrm{NPos}\right)\times 100 $$

Subsequently, the resultant value in the baseline condition was subtracted from each of the four main conditions. As such, the negative conditional means illustrated in Fig. 2a represent lower accuracy than in the baseline condition (1Team), with more negative values indicating poorer relative performance. The omnibus ANOVA on the relative data revealed a main effect of CountTask, *F*(1, 28) = 26.759, *MSE* = 10.874, *p* < .001, η_p_^2^ = 0.489, with better performance in the CountAll (*M* = −0.743) versus CountTeam (*M* = −3.91) conditions, a main effect of NTeams, *F*(1, 28) = 6.735, *MSE* = 2.143, *p* = .015, η_p_^2^ = 0.194, with a better performance in the 2Teams (*M* = −1.974) condition versus 5Teams (*M* = −2.679) condition, and a significant interaction, *F*(1, 28) = 5.348, *MSE* = 1.964, *p* = .028, η_p_^2^ = 0.160. Planned paired comparisons between performance for 2Teams and 5Teams in both CountAll and CountTeam tasks did not show evidence of a difference in performance for 2Teams (*M* = −0.691) versus 5Teams (*M* = −0.794) in the CountAll condition (*p* = .66), but confirmed that participants were significantly more accurate in counting the number of possessions within a given color team when there were two teams of 10 players each (CountTeam2Teams; *M* = −3.257) versus when there were five teams of four players each (CountTeam5Teams; *M* = −4.564), *t*(28) = 2.732, *SEM* = 0.479, *p* = .011, Cohen’s *d* = 0.41. A final set of one-sample *t* tests confirmed that both CountTeam2Teams and CountTeam5Teams conditions of interest were significantly different from baseline, both *t*s(28) > 5.981, both *p*s < .001, both Cohen’s *d*s ≥1.1, and that both CountAll2Teams or CountAll5Teams conditions were marginally significantly different from baseline (1Team) performance, both *t*s(28) > 1.91, both *p*s < .066, both Cohen’s *d*s ≥0.355.

### Pupil diameter

We also calculated the average relative pupil diameter for each subject in each condition by dividing each participant’s average pupil diameter in a given condition by their corresponding average pupil diameter over all trials.^2^ If the pupil was lost for 400 ms or less (e.g., during blinks), pupil diameter was linearly interpolated from the four samples immediately before and after the pupil was lost. The data in Fig. 2b represent average relative pupil diameters in all four CountTask × NTeams conditions, with positive values indicating larger average relative pupil diameters than in the baseline (1Team) condition. The main ANOVA revealed a significant main effect of CountTask, *F*(1, 28) = 15.14, *MSE* = 0.001, *p* = .001, η_p_^2^ = 0.351, with smaller relative pupil diameters in the CountAll (*M* = 0.014) versus CountTeam (*M* = 0.038) conditions, a significant main effect of NTeams, *F*(1, 28) = 11.223, *MSE* = 0.001, *p* = .002, η_p_^2^ = 0.286, with smaller relative pupil diameters in the 2Teams (*M* = 0.018) versus 5Teams (*M* = 0.034) conditions, and most importantly, a significant interaction between these factors, *F*(1, 28) = 5.075, *MSE* < 0.001, *p* = .032, η_p_^2^ = 0.153. Planned paired comparisons between the 2Teams and 5Teams conditions in both *CountAll* and *CountTeam* tasks did not show evidence of a difference in relative pupil diameter for 2Teams (*M* = 0.009) versus 5Teams (*M* = 0.019) in the CountAll condition (*p* = .108), but confirmed that participants had significantly smaller pupil diameters in the CountTeam2Teams condition (*M* = 0.027) compared with the CountTeam5Teams condition (*M* = 0.049), *t*(28) = 4.429, *SEM* = 0.005, *p* < .001, Cohen’s *d* = 0.521. The final set of one-sample *t* tests confirmed that average relative pupil diameters in the four conditions were significantly different from pupil diameter in the baseline condition, all *t*s(28) ≥ 2.54, all *p*s ≤ .018, all Cohen’s *d*s ≥ 0.474.

## Discussion

Overall, results confirmed our main hypothesis that observers would be better able to track the interactions of a team of players when there were fewer teams but more individual players per team. These results suggest that grouping by similarity (i.e., color) facilitates performance at the group level, regardless of the number of individual elements in the group. Contrary to theories that predict observers will be worse at tracking the interactions between multiple elements as set sizes increase (e.g., FINST; Pylyshyn & Storm, 1988), observers were more accurate and the task was less demanding when they were counting the possessions of a prespecified team and there were only two teams of 10 players each, compared with when there were five teams of four players. This conclusion is supported by the interactions between CountTask and NTeams, taken with the significant differences between the 2Teams and 5Teams conditions in the CountTeam task for both percentage correct and pupil diameter measures. These patterns of results are also in line with recent proposals that Gestalt grouping reduces the resources necessary to monitor individual objects, allowing more objects to be processed when they belong to the same group (Brady & Tenenbaum, 2013; Corbett, 2017; Gao et al., 2011; Peterson et al., 2015; Woodman et al., 2003; Xu & Chun, 2007), and further suggest that this facilitation occurs across all items in the set rather than at the level of individual objects (Scholl, 2001; Yantis, 1992). Finally, whereas Porter et al. (2007) reported that pupil size increased with search difficulty and set size, the present results suggest that changes in pupil dilation are not necessarily a function of the number of items. Instead, our findings of smaller pupil diameters in the CountTeam2Teams versus CountTeam5Teams condition suggest that grouping reduced the mental effort required to track the ball when there were more individuals on fewer teams despite the increase in set size.

In addition to our main hypothesis, we also predicted that observers might rely on grouping arrangements differently, depending on their top-down set (i.e., whether they were instructed to count the possessions by all of the players or only by players on a specific team at the start of each trial). For example, knowing in advance that they should pay attention to the possessions of all the players regardless of team membership may have biased observers to monitor the position of the soccer ball differently than when they knew in advance that they would only have to count the possessions of a specific team (and they could “ignore” possessions by all other teams). Along these lines, there were main effects of CountTask on accuracy and pupil diameter, with superior performance in the 1Team baseline condition. In line with converging reports that parsing displays into subsets comes with a cost (Attarha & Moore, 2015; Attarha et al., 2014; Brand et al., 2012; Oriet & Brand, 2013; cf. Chong & Treisman, 2005), these results suggest that there is a similar “cost” when tracking the interactions of subsets within a larger display of objects.

Although the present study clearly demonstrates that grouping by similarity facilitates tracking the interactions between individual objects at the set versus object level, several limitations must be considered when interpreting the observed effects. First, we initially tested 20 participants (the data from four of these participants were excluded from analyses based on performance or equipment issues outlined in the Method section) based on sample sizes used in similar previous research (e.g., Alvarez & Franconeri, 2007; Pylyshyn, 2004; Scholl, 2001). However, as the results of interest were only marginally significant, we opted to approximately double our sample size. Although we note that this two-stage analysis does increase the risk of reporting false positives in the present results, we also wish to point out that there is currently no straightforward method for an a priori computation of the sample size necessary to observe main and interaction effects of prespecified sizes with a given level of power (typically 0.8) in repeated-measures within-subjects designs such as ours. One potential solution would have been to repeat the entire experiment after we had obtained the present significant patterns of effects with 39 total participants (with 10 of these participants excluded from analyses). However, this was not possible given financial, physical, and temporal constraints. Second, we note that our results are limited in their generalizability to real-world situations. Obviously, there are substantial differences between our displays of simple two-dimensional stimuli with randomized movement patterns and players in an actual soccer match, especially with regard to how players’ “body language” and other social cues may additively influence observers’ abilities to track possessions of the ball. Therefore, future investigations using videos of actual soccer players can help to better understand how such additional social factors may affect our abilities to track the interactions in real-world situations. For example, comparing between performance obtained using such real-world displays and performance using displays with our simple shapes superimposed on players to retain only their movement patterns can help to better understand the potential contributions of social exchanges in real-world interactions. In addition, we investigated whether grouping improves the ability to track interactions of more individual players or whether facilitation occurs at the level of the entire group of players by comparing performance with a larger number of groups of fewer players to performance with fewer groups of more players each. Such investigations require that the total number of objects in each display (20 in the present study) remains constant across different conditions. Unfortunately, this makes it impossible to systematically manipulate the number of groups independently of the number of players in each group. The present findings do establish initial evidence that grouping facilitates processing for fewer groups of more individual objects, paving the way for comparisons with future studies independently manipulating the number and size of different groups. Finally, although we have repeatedly referred to the multiple object tracking literature, we acknowledge that our task is not a multiple object tracking task, per se. Instead of having to keep track of the locations of a number of stimuli, observers in our task simply had to track one stimulus (the ball). Nonetheless, we observed a strong influence of grouping, such that observers were better able to track the ball when there were fewer groups of more objects versus when there were more groups of fewer objects each.

In sum, our results provide further support for previous proposals that grouping facilitates observers in tracking the interactions between multiple objects, and further suggest that this facilitation is spread across the entire set instead of acting at the level of individual objects. The current findings can also help to inform techniques for improving sports performance by practicing multiple object tracking tasks in the lab (e.g., Romeas, Guldner, & Faubert, 2016). Overall, the present study aligns with proposals that the limited capacity visual system relies on grouping heuristics in a flexible, task-driven fashion to efficiently represent the dynamic external environment.

## Footnotes

1 Note that we did not compare measures of fixations or saccades because the 60-Hz sampling rate of our equipment introduced as much as 16.67 ms of noise in individual samples.

2 Pupil diameter was recorded in ASL’s native “Eye Image Coordinates.”
